# A comprehensive procedure for antiviral inhibitor discovery using EV71 as an example

**DOI:** 10.1007/s41048-015-0006-z

**Published:** 2015-08-22

**Authors:** Lin Cao, Shouhai Zhu, Yaxin Wang, Zhiyong Lou, Yuna Sun

**Affiliations:** College of Biotechnology, Tianjin University of Science and Technology, Tianjin, 300457 China; National Laboratory of Macromolecules, Institute of Biophysics, Chinese Academy of Science, Beijing, 100101 China; Laboratory of Structural Biology, School of Medicine, Tsinghua University, Beijing, 100084 China

**Keywords:** Virus, Antiviral, Entry, Replication, Assay

## Abstract

The prevalence of chronic viral infectious diseases, the emergence and re-emergence of new viral infections, and in particular, resistance to currently used antiviral drugs have led to increased demand for new antiviral strategies and reagents. Increased understanding of the molecular mechanisms of viral infection has provided great potential for the discovery of new antiviral agents that target viral proteins or host factors. In this work, we introduce a comprehensive system using enteroviruses 71 (EV71) as an example for leading compound discovery to develop new antiviral.

## INTRODUCTION

Viruses constitute a large family of pathogens to cause severe infectious diseases throughout human, animal, plant and bacteria. Over the past decades, antiviral agents that target viral proteins or host factors have been successfully developed. Based on their inhibitory mechanisms, antiviral reagents can be divided into three groups, i.e., virus-targeting antivirals (VTAs), which directly (DVTAs) or indirectly (InDVTAs) inhibit the biological function of virally encoded proteins, and host-targeting antivirals (HTAs) that inhibit the function of host protein which are utilized by virus in the life cycle (Lou et al. 2013; Zhou et al. 2013).

With increased knowledge of viral protein and host factors, great process has achieved significantly progress in the discovery against chronic viral infectious diseases, the emergence of new virus caused diseases, and the resistance to traditional antivirals. In the early stage of antiviral development, the phenotype-based screening was launched to find reagents that can inhibit the proliferation of virus in the cell-based assays. With the increasing knowledge of structural biology and virology, the understanding of the molecular mechanism of viral proteins or host factors that can be utilized by virus for the efficient multiplication allowed the researchers to design the compounds those can be fitted into the active site of target protein. In particular, the fragment-based drug discovery can lead people make new compound from several fragments binding in different position of the active site of target protein. However, in the last 10 years, the target-based strategy was challenged. The difficulties on compound synthesis, low bioavailability, poor pharmacokinetic property, and toxicity result in the low ratio of success. Meanwhile, poor biology, lack of disease relevant assay, poor target validation, and lack of biochemical information also lead to the failure of target-based strategy. Therefore, the phenotype-based screening returned to the main track of antiviral discovery in recent years and led to several great successes in the development of reagents to treat HCV, HIV-1, Dengue, and other viral infections. In this work, we used EV71 to present a comprehensive system for the phenotype-based discovery of leading compound against viral infection.

## OVERALL DESCRIPTION OF ANTIVIRAL INHIBITOR DISCOVERY SYSTEM

**Fig. 1:**
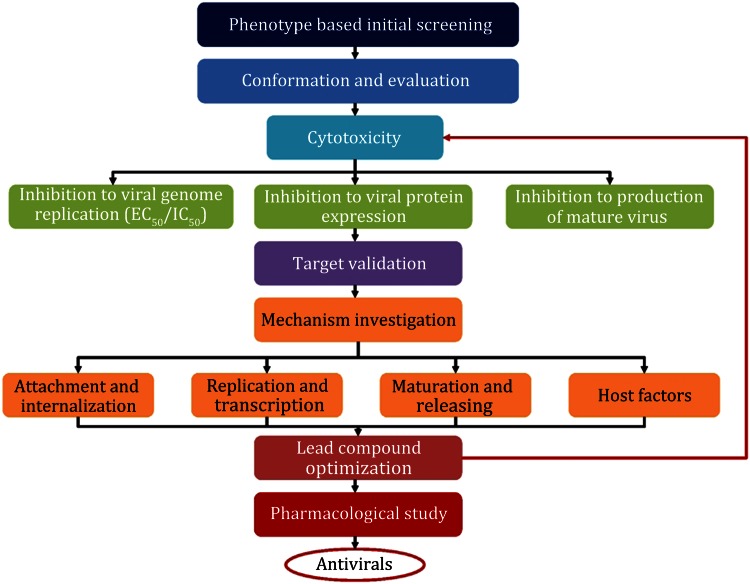
Workflow for a phenotype-based antiviral discovery system

A phenotype-based antiviral discovery pathway can be generally divided into the early discovery of potential compound, inhibition effect conformation and evaluation, target discovery, mechanism investigation, compound optimization, and further pharmacological research (Fig. 1). The general protocol presented here has been used successfully by our group for the discovery of several antiviral leading compounds with high potency. Because the compound optimization and pharmacological research are in very late stage, we focus on the early stage of antiviral discovery in this work.

## RESULTS

### Phenotype screening to discovery potential inhibitor

**Fig. 2:**
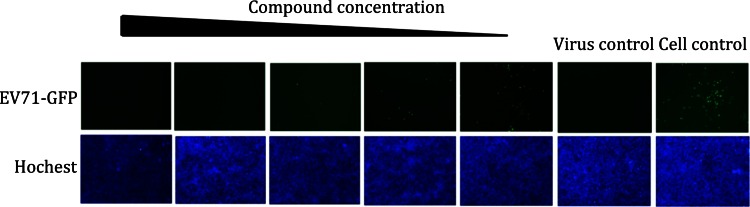
A GFP-based initial screening assay used for anti-EV71 reagent development. Compounds were added to the RD cells in a concentration series from high to low and infected by the virus. After 24 h, the expression of GFP (*top panel*) and the nuclei dyed by Hoechst (*bottom panel*) were observed under microscope to define the effective compound concentration

We performed the initial screening through a GFP-based assay (Fig. 2). The intensity of GFP was observed under the microscope to determine the antiviral activity of compound (Fig. 2, upper panels). As the control, Hoechst is usually used to visualize the nuclei, marking up the host cells, to monitor the virus growth (Fig. 2, lower panels).

In an initial screening, we select the compounds, which can protect the host cell against virus infection (e.g., 50% host cells can survive) at the concentration lower than 20 µmol/L, for our further validation. However, this limitation is not a strong cap. It is highly dependent on the type of virus and cell lines.

### The security of inhibitors on cell level

**Fig. 3:**
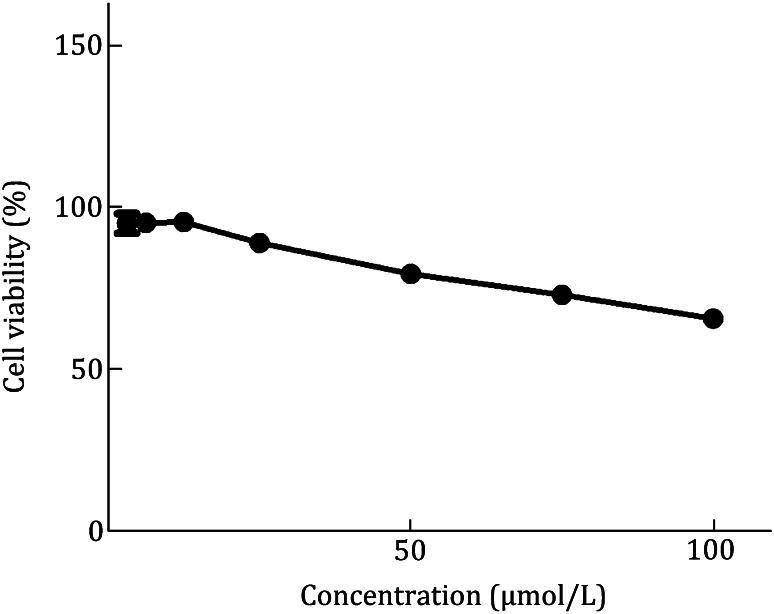
The measure of cytotoxicity of a compound. To monitor cytotoxic effect, the viability cells following compound treatment were determined using WST-1 based assay and compared to that of untreated control cells at 24 h post infection (hpi)

Because the compounds can occasionally hurt host cells and result in false positives, we would like to check the cytotoxicity of the compounds selected from the initial screening to demonstrate the specificity of the inhibition. In general, we test the cytotoxicity by using the cells which support the virus growth, but we also use Vero cell as a general control to monitor the cytotoxicity of these compounds. There is no obviously cytotoxicity of compound on RD cells (Fig. 3).

If the CC_50_ value is much higher than the concentration that can inhibit virus proliferation of approximately 20-fold, we believe that this inhibition to virus growth is specific and will subsequently measure the inhibition to the replication of viral genome, the expression of viral protein, and the production of mature virus.

### The inhibitory activity of compound

**Fig. 4:**
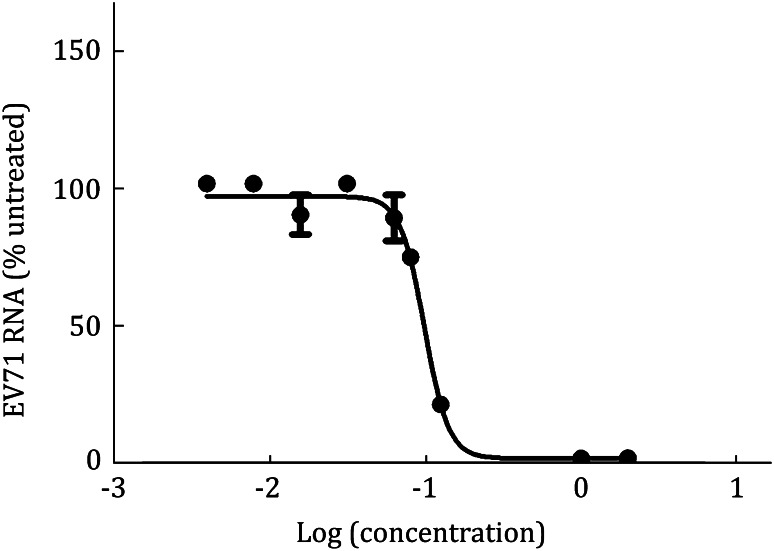
*EC*_50_/*IC*_50_ measured by qRT-PCR method. The levels of EV71 RNA were quantified by qRT-PCR at 24 hpi and were expressed as the percentage of the level of EV71 RNA for the untreated control cells (the mRNA level of GADPH as an internal reference). Each data point represents the average for three replicates in a single experiment

We further studied the anti-EV71 activity of compound by infecting RD cells with EV71 virus. The infected cells were treated with various concentrations of compound for 24 h, and the remaining levels of EV71 RNA in RD cells were determined by qRT-PCR. The results revealed that the tested compound showed a concentration-dependent reduction of EV71 RNA in the infected cells (Fig. 4).

### Western blot analysis

**Fig. 5:**
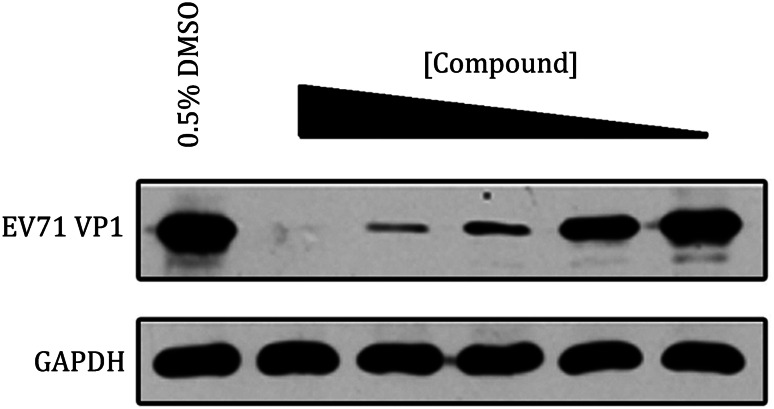
Western blot analysis to monitor the change of the expression of viral protein. The inhibition of EV71 VP1 expression treated with different concentrations of compound, comparing to the expression of an internal control, GADPH

We used western blot to monitor the inhibition of virally encoded protein by compounds. The result showed that the inhibitor was dose-dependent and the expression of EV71 VP1, which is the major component of EV71 capsid, was obviously attenuated by the treatment of suramin. In a contrast, the expression of endogenous glyceraldehyde-3-phosphate dehydrogenase (GADPH) was not affected, being indicative that the inhibition of this inhibitor to EV71 replication was specific (Fig. 5).

### Cell binding assay

The entry stage of EV71 infection can be further subdivided into two steps: (1) the attachment of EV71 virion to host cell mediated by functional receptors and (2) uncoating to release viral genome into host cell. To further define the inhibitory mechanism of compound on EV71 entry, we analyzed the binding affinity of EV71 virion to host cell with the treatment of compound.

**Fig. 6:**
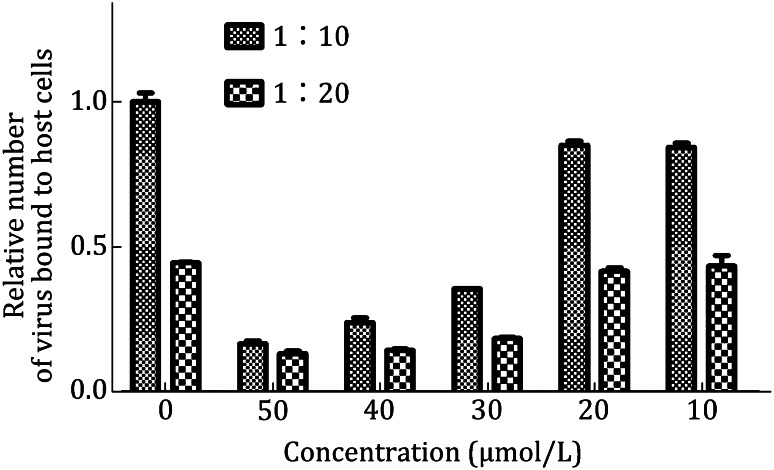
Cell binding assay. RD cells were used to compare the cell binding capacity of EV71 under the treatment with inhibitor at different concentration (50, 40, 30, 20, and 10 µmol/L, respectively). Two conditions were assessed: tenfold diluted (1:10) and 20-fold diluted (1:20) standardized viral stocks (10^8^ TCID_50_/mL). The quantification of bound virus was measured by quantitative real-time RT-PCR assay and expressed as the virus binding by using 0.5% DMSO as control. Vertical bars indicate minimum/maximum values

We incubated RD cells with EV71 virus with the titer of tenfold diluted (1:10) and 20-fold diluted (1:20) standardized viral stocks in the absence or presence of compound. In the presence of 50 μmol/L compound, the virus bound to host cell in two conditions decreased to less than 20% of that seen in the absence of compound. When the concentration of compound dropped to 20 μmol/L or 10 μmol/L, the bindings to host cell were both rescued. These results revealed that compound directly blocked the attachment of EV71 virion to host cell (Fig. 6).

### Immunofluorescence assay (IFA)

**Fig. 7:**
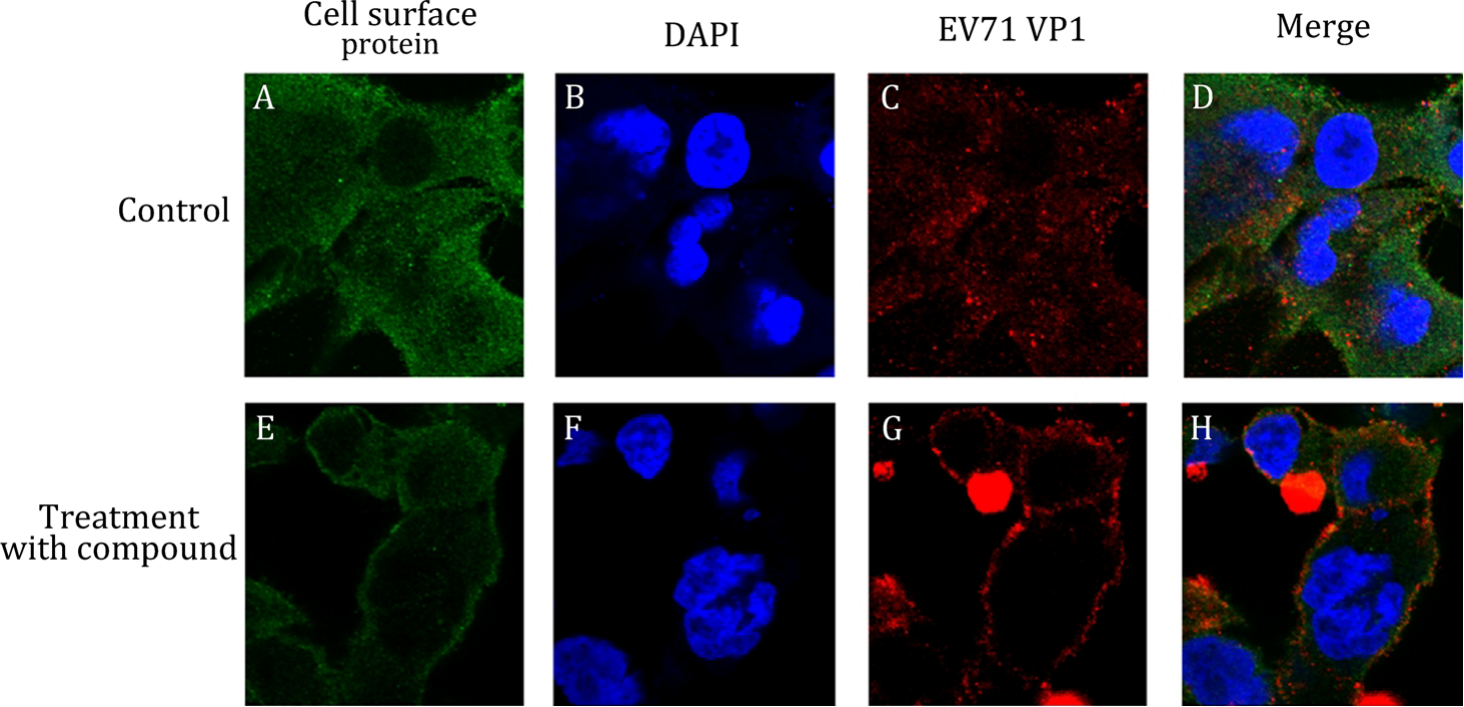
Immunofluorescence assay (IFA). Immunofluorescence analysis was performed on RD-sh-control and RD-sh-cell surface protein cells infected with EV71 at a MOI of 50. After 2 hpi, cells were fixed and stained with anti-cell surface protein (*panels* **A** and **E**, *green*) and anti-EV71 VP1 (*panels* **C** and **G**, *red*) antibodies, and DAPI was used to visualize the nuclei (*panels* **B** and **F**, *blue*). *Panels* **A**–**D** and **E**–**H** show the same cells. Merged images are shown in *panels* **D** and **H**, respectively

We further examined the internalization of EV71 by using immunofluorescence (Fig. 7). The RD-sh-control and RD-sh-cell surface protein cells were infected with EV71, and endogenous protein and EV71 VP1 proteins were analyzed by immunofluorescence. In the RD-sh-control cells infected with wt-EV71, EV71 VP1 was distributed throughout the cytoplasm at 2 hpi, which was indicative that the virus particle internalization and localization with cell surface protein was random (Fig. 7, panel A–C). In the downregulation of cell surface protein in the RD-sh-cell surface protein cells infected with wt-EV71, the localization of wt-EV71 was restricted to the cytoplasm of the perimembrane region at 2 hpi (Fig. 7, panel E–G). We can also observe the colocalization of EV71 VP1 with cell surface protein, suggesting that the knockdown of cell surface protein inhibited the internalization of EV71 and cell surface protein was accumulated around EV71 virions.

### In vitro enzymatic assay: protease

**Fig. 8:**
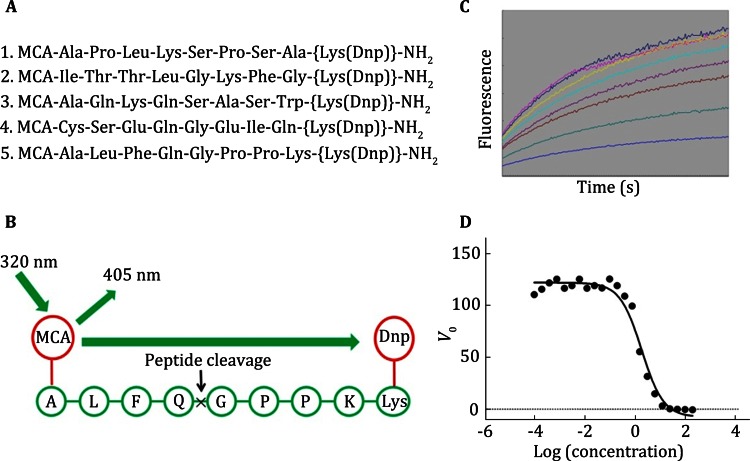
In vitro enzymatic assay—protease. *IC*_50_ of the peptidomimetic inhibitors against the EV71 3C protease. **A** Five synthetic peptides used in enzymatic assay. **B** Principle of fluorescence resonance energy transfer (FRET). **C** *Curves* of different inhibitors at series concentrations and times. **D** The calculation of *IC*_50_ value of one inhibitor

We studied the anti-EV71 3C^pro^ activity of compound. The 3C^pro^, the best fluorogenic peptide and compound incubate a few minutes in buffer. The initial velocities of the enzymatic reactions (within the first 5 min) were determined and fitted to a sigmoidal dose-response equation with nonlinear regression analysis using the program GraphPad Prism 5 software. The data from three independent assays were used as input for Prism to calculate the *IC*_50_ and 95% confidence interval values (Fig. 8D). The results revealed that the tested compound showed a concentration-dependent reduction of 3C^pro^.

### In vitro enzymatic assay: polymerase

**Fig. 9:**
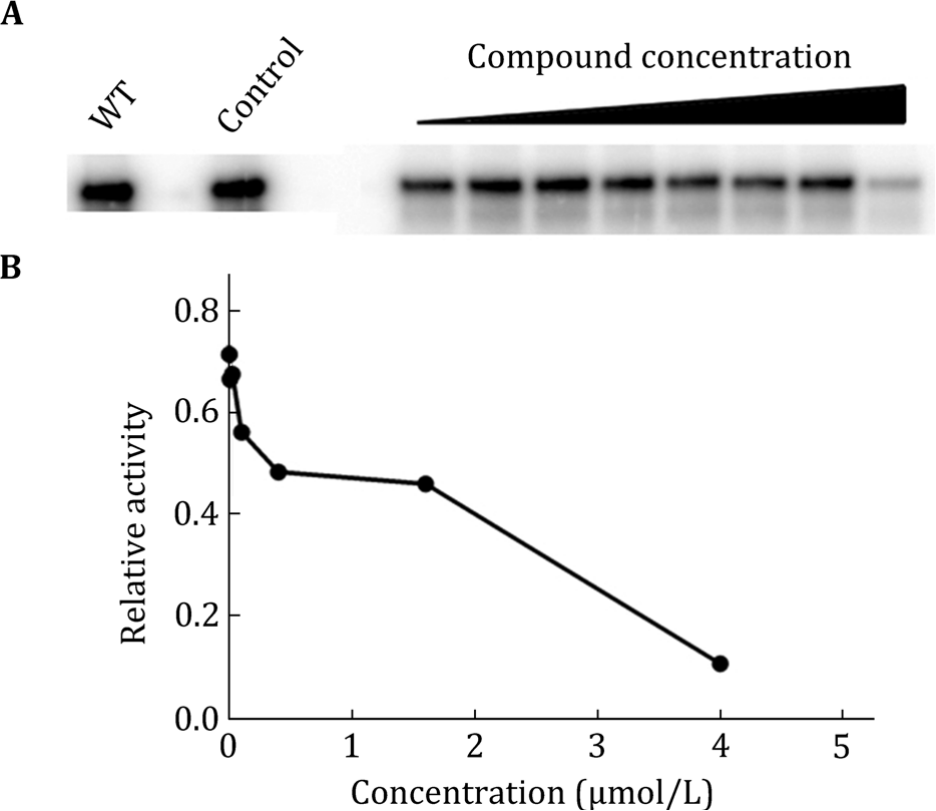
In vitro enzymatic assay—polymerase. **A** SDS-PAGE of the reaction products treated with different concentration of compound. **B** The relative activity of polymerase under increasing concentration of compound

An in vitro assay was used to test whether compound functions as a chain terminator to inhibit EV71 genome replication. This biochemical approach used a primer extension-based RdRp assay. This assay measures the incorporation of [α-33P] ATP in the presence of ATP using polyU as the RNA template. A triphosphate derivative of compound (ppp-compound) was chemically synthesized to serve as an RdRp substrate. Addition of ppp-compound to the RdRp reaction reduced the amounts of RNA products in a dose-dependent manner. The results suggested that ppp-compound acts on viral RdRp to terminate RNA synthesis (Fig. 9).

## METHODS

### Cell lines, viruses, and reagents

Stable cell lines are essential for antiviral discovery. To evaluate the inhibition effect, we select one stable cell line for the initial screening, and two or three cell lines for further validation. For example, we usually use RD (human embryonal rhabdomyosarcoma) cell lines for anti-EV71 drug discovery (Chen et al. 2013; Sun et al. 2012). RD cell is a commercially supplied cell line and we purchased it from ATCC Company. We culture RD cell in Dulbecco’s modified Eagle’s medium (DMEM) (GIBCO) supplemented with 10% fetal bovine serum (FBS) (GIBCO) at 37 °C in a humidified incubator with 5% CO_2_. After the initial screening, we additionally use the kidney cell of African green monkey (Vero) to further validate the inhibition effect. Vero cell is cultured under the same condition of the culture of RD cell. Although the cell lines for virus proliferation are highly dependent on the type of virus, Vero cell has also been widely used in the detection of chemical’s toxicity.

To evaluate the inhibition effect to various virus stains, we use two or three virus stains for the entire pipeline, including a prototype stain and one or two clinical strains. For example, we use the prototype strain BrCr (U22521) and a clinical strain AnHui1 (GQ994988.1) of EV71 in our research. For initial screening, we would like to insert a GFP reporter gene into the genome of target viral genome. For GFP and reporter virus, the fluorescent gene was inserted between the 5′UTR and the N-terminus of VP4 gene with the addition of an EV71 2A protease cleavage site (ITTLG) at its C-terminus. Upon the cleavage by 2A protein, the fluorescent protein is released and visible under a fluorescent microscope. The GFP expression in the GFP-EV71 reporter virus-infected cells correlates with viral RNA replication. The wild-type (wt) and GFP-inserted virus are amplified and quantified by the determination of the 50% tissue culture infective dose (TCID_50_) per 1 mL in host cells as previously described (Lin et al. 2002), and are used for all experiments.

The compounds for screening are permanently stored as powder in −80 °C and were initially dissolved in dimethyl sulfoxide (DMSO) with a certain concentration (we prefer 10 mmol/L), and stock solutions were stored at −20 °C. Immediately before addition, these compounds were diluted to the desired concentrations using DMEM with 10% FBS. However, a key issue is often ignored that the compound which is stored in DMSO at −80 °C may have degradation during long-term storage. This caused several unexpected observations in our research (unpublished result). We suggest that the compounds which are stored in DMSO solution should be updated one or two years to avoid degradation.

#### Phenotype screening

In the discovery of anti-EV71 reagent, we first seed 3 × 10^4^ RD cells per well in 96-well microtiter plates 24 h before experiment and then supply the compounds after serially diluted compounds in the presence of 10% FBS and 0.5% DMSO to the desired concentration. After adding compounds, 100 × TCID_50_ virus is used to transfect RD cells for 24 or 48 h, and then we observed the GFP under microscope.

#### Cytotoxicity

To monitor the cytotoxic effect of the compounds selected from anti-EV71 screening assay, the viability of RD or Vero cells following 24 h of serially diluted compounds in the presence of 10% FBS and 0.5% DMSO treatment was determined in 96-well tissue culture plates using cell proliferation reagent WST-1 (Roche). Cells are seed at a concentration of 3 × 10^4^ cells/well with additional compounds at various concentrations (0.1–200 µmol/L) and are incubated for another 24 h. After that, WST-1 reagent is supplied to each well for an incubation of 4 h before shaking thoroughly for 1 min on a shaker. We can consequently measure the absorbance of the samples at 420–480 nm using a microplate reader.

The viability of cells is plotted by GraphPad Prism 5 software and the percentage of cytotoxicity was calculated as follows:  % cytotoxicity = (1 − (average of compound-treated cells)/(average of control cells)) × 100. The concentration that 50% cells can survive (*CC*_50_) is calculated in GraphPad Prism 5 software.

#### Measure *EC*_50_/*IC*_50_

We usually measure the concentration for 50% of maximal effect (*EC*_50_) or 50% of maximal inhibition (*IC*_50_) through quantitative RT-PCR (qRT-PCR) method to monitor the changing of viral genomes. In our anti-EV71 assay, 10^5^ RD cells are seeded in each well of 24-well tissue culture plates and were allowed to attach in complete culture medium overnight. The culture medium was replaced with medium containing serially diluted compounds in the presence of 10% FBS and 0.5% DMSO. After adding compounds, we use 100 × TCID_50_ virus to transfect RD cells. After the cells were treated for 24 h, total cellular RNA was isolated by Trizol reagent using standard protocols. qRT-PCR assay (for primer sequences, GADPH as an internal control, forward primer 5′-CCC ACT CCT CCA CCT TTG ACG-3′ and reverse primer 5′-CAC CAC CCT GTT GCT GTA GCCA-3′, EV71 5′UTR forward primer 5′-TGA ATG CGG CTA ATC CCA ACT-3′ and reverse primer 5′-AAG AAA CAC GGA CAC CCA AAG-3′) was performed by QuantiTect SYBR Green RT-PCR kit (QIAGEN, Valencia, CA) as manufacturer protocol. EV71 and GAPDH transcript levels were determined by ∆∆CT method (Schmittgen and Livak 2008). The percentage of inhibition was calculated as follows:  % inhibition = (1 − (average of compound-treated cells)/(average of control cells)) × 100. *IC*_50_ is the concentration of compound at which the EV71 RNA level in the RD cells is reduced by 50%. The values of *EC*_50_ or *IC*_50_ are plotted by the GraphPad Prism 5 software.

#### Western blot analysis

To monitor the inhibition of virally encoded protein by compounds, we infect the host cells with virus for 24 or 48 h. Cells are then lysed in a buffer containing 50 mmol/L Tris-HCl, pH 8.0, 150 mmol/L NaCl, 1% NP-40, 0.5% sodium deoxycholate, 0.1% sodium dodecyl sulfate (SDS), 2 mmol/L EDTA, 1 mmol/L NaVO_4_, 10 mmol/L NaF, and protease inhibitors, and the protein concentration in the lysates was determined by a spectrophotometer. Proteins are resolved by sodium dodecyl sulfate polyacrylamide gel electrophoresis (SDS-PAGE) and transferred of proteins from gel to NC membrane (Millipore). NC Membranes were blocked for 4 h with 5% nonfat dry milk solution in Tris-buffered saline and as thorough removal of the unbound reagents after each of the binding steps in the procedure. This is terminated by performing series of washes, and then blotted with specific primary antibodies, wash away excess primary antibodies, and incubate with secondary antibodies conjugated with horseradish peroxidase. Proteins were visualized by chemiluminescence and clarity western ECL substrate (BIO-RAD) (Fig. 5).

#### Cell binding assay

Cell binding assay can help us to direct measure the viral attachment to host cells with or without the treatment of compounds. In our anti-EV71 reagent discovery system, RD were seeded at 1 × 10^5^ RD cells/well in 24-well plates. After 24 h culture, medium was removed and cells were washed once with cold phosphate buffer saline (PBS). 500 μL of binding buffer (PBS containing 1% BSA and 0.1% sodium azide) was added to cell on ice for 10 min and the supernatant was subsequently removed from cells. The 10^8^ TCID_50_/mL of virus was prepared. 500 μL of EV71 virus diluted by DMEM complete medium (dilution fold = 1:10 or 1:20) with 0.5% DMSO or additionally compound was added. After 1 h of incubation on ice, unbound virus was removed by three wash steps with 500 μL PBS and then cells were lysed in the wells with 500 μL Trizol reagent. Viral RNA was extracted and detected by qRT-PCR (Fig. 6). The virus binding assays were performed systematically in duplicate in two individual experiments for each condition to avoid false positives.

### Target discovery

In the structure or target-based antiviral development, the target for inhibitor action is clear. But a key question for the phenotype-based antiviral discovery is what the exact working target is for antiviral function. This is also critical for the further investigation of inhibition mechanism and the optimization of the inhibitors. The most potent method for the identification of working target is screening of drug-resistant virus. Since the virus mutates its genome and protein at specific position where the antiviral acts to conquer the drug treatment, sequencing of the entire viral genome to identify the mutation helps us to know the working target of the potential inhibitors.

In our anti-EV71 drug screening assay, RD cells were seeded at 5 × 10^5^ cells/well in 6-well plates. After 24 h culture, the media were removed and replaced with DMEM containing 10% FBS and the inhibitor (the inhibition is above 90%), 0.5% DMSO as control. After 8 h, virus stain was used to infect RD cells at 0.1 MOI in media containing inhibitors. During the course of selection, RD cells were split when a 70%–90% confluence was reached. Fresh media containing inhibitors were added when the cell culture was split. The virus replication in the presence of inhibitors was monitored by CPE at each passage. The viruses demonstrate apparent cytopathogenic effect (CPE) about 5 to 7 days in media containing inhibitors or 2 days in media containing 0.5% DMSO after virus infection. Then the cell supernatants were collected following centrifugation at 4000 *g* for 5 min and were stored at −80 °C as Virus-P2 (passage 2) virus. Then the RD cells were treated with inhibitors for 8 h and infected with Virus-P2 virus at the same condition as above. Until the experiment was cycling operated for six times, and the cell supernatant was collected as Virus-P6 (passage 6) virus. The RD cells were lysed by using Trizol reagent.

For EV71 RNA resistance mutation analysis, intracellular RNA extraction was performed by using Trizol reagent (Invitrogen) according to the manufacturer’s instructions. For reverse transcription PCR, the first-strand cDNA was synthesized by using a gene specific primer (5′- ACCCCCACCAGTCACATTCACG-3′) and Super Script III First-strand Synthesis System for RT-PCR kit (Invitrogen) according to the manufacturer’s instructions. The short RT-PCR products of the resistant EV71 virus or control EV71 virus were ligated into the TA cloning vector PMD18-T (Takara). For each time point, multiple individual bacterial colonies were isolated, and the purified plasmid DNA was sequenced. The most mutated sites are very likely to be the drug working sites and we can test whether the targets are identified through drug-resistance screening further.

To confirm, the mutant was approximately over fivefold higher than the *EC*_50_ value for wt-EV71.

### Mechanism investigation

Once the working targets of inhibitors can be defined by the sequencing of drug-resistant viruses, we would like to further characterize the inhibition mechanism through virology and biochemical methods. The virus life cycle can be simply featured into the entry, replication and transcription, and maturation and releasing processes. If the inhibitors function at the entry step, we use cell binding assay and immunofluorescence assay to check the binding affinity between virus and host cell or functional receptors. If the inhibitors function at the replication and transcription step, the in vitro enzymatic assays are preferred. We present a general protocol which we use in anti-EV71 drug screening work.

#### Immunofluorescence assay (IFA)

We can further directly view the binding of virus to host cells through IFA assay. RD cells were grown on cover slips till 50% confluency and infected with EV71 virus. At the indicated times post infection, cells were washed with PBS and fixed with 4% paraformaldehyde for 15 min at room temperature, washed, permeabilized with 0.5% Triton X-100 in PBS for 10 min, washed, and blocked with 1% normal goat serum in PBS for 30 min, followed by a 1 h incubation with primary antibodies 1:400 at room temperature. After three washes with PBS, cells were incubated with FITC- or PE-conjugated secondary antibodies at a 1:200 dilution for 1 h. After extensive washing with PBS, cell nuclei were stained with DAPI. Images were captured by confocal microscopy (Olympus FluoView FV1000 Confocal Microscope operated by FluoViewTM software) (Fig. 7). If the treatment of compound obviously causes the accumulation of virus at the host membrane region, this compound is believed to inhibit the entry of virus.

#### In vitro enzymatic assay: protease

The inhibition of compound to viral transcription and replication step usually works at viral protease or polymerase activity. To detect the inhibition to viral protease activity, we need an in vitro cleavage assay. There are five peptides in the experiment (Fig. 8A), and the cleavage assay was performed in a reaction volume of 200 to yield a final concentration of 5 μmol/L enzyme and 500 μmol/L peptide, residual volume filling with buffer. Cleavage reactions were routinely incubated at 25 °C for 5 min and then terminated by the addition of an equal volume of 2% trifluoroacetic acid. The samples were analyzed by reverse-phase high-performance liquid chromatography (HPLC) on a C18 column (4.6 by 250 mm) using a 20-min, 5%–45% linear gradient of acetonitrile in 0.1% trifluoroacetic acid. The absorbance was monitored at 215 nm. The area of the product peak was calculated and transformed into a product-peptide concentration, based on which the efficiency of each peptide being processed by protease could be determined. The cleavage efficiency was defined as the average rate within 5 min of a selected peptide being cleaved by 5 μmol/L either protease with an initial peptide concentration of 500 μmol/L. Then the best peptide substrate was attached with a fluorescence quenching pair, and the fluorogenic peptide. It was used as the substrate for the inhibition assay. This peptide contains the proteinase site, with excitation at 320 nm, and enhanced fluorescence could be observed at 405 nm after the cleavage of the peptide (Fig. 8B). This enabled us to monitor the peptide cleavage in real time (Fig. 8C). The enzyme concentration used in measuring *K*_m_ and *K*_cat_ values was 0.5 μmol/L and the substrate concentrations were from 0.5- to 5-fold of an estimated *K*_m_ value. Substrate concentration was determined by using the extinction coefficients 5438 L/(mol·cm) at 336 nm (Edans) or 15,100 L/(mol·cm) at 472 nm (Dabcyl). The initial rates within 10% substrate consumption at different substrate concentrations were used to calculate the kinetic parameters using Michaelis-Menten equation fitted with the KaleidaGraph computer program.

In our anti-EV71 assay, to determine the 50% inhibitory concentrations (*IC*_50_) of inhibitors against 9 μmol/L protease, 150 μmol/L fluorescent peptide, and gradient concentrations of inhibitor were mixed in a buffer. The initial velocities of the enzymatic reactions (within the first 5 min) were determined and fitted to a sigmoidal dose-response equation with nonlinear regression analysis using the program GraphPad Prism 5 software. The data from three independent assays were used as input for Prism to calculate the *IC*_50_ and 95% *CI* values (Fig. 8D).

#### In vitro enzymatic assay: polymerase

The inhibition to viral polymerase activity can be evaluated by measuring the extension of viral genome. For example, in our anti-EV71 drug discovery system, the replication process of EV71 presents a template-dependent manner, by using a small stem loop structure (*cis*-acting replication element, CRE) as the natural template and the protein VPg as a primer(Chen et al. 2013; Sun et al. 2012). For the RdRp assay, the uridylylation assay mixture (20 μL), containing 50 mmol/L HEPES (pH 7.5), 0.5 mmol/L magnesium chloride, 7% glycerol, 1 μg of CRE, 50 μmol/L VPg, 1 μg of EV71 3D, and 1 μCi [α-^32^P]UTP, was incubated at 30 °C for 1 h. After the reaction, the aliquots were mixed with the same volume of 2 × Tris-Tricine SDS buffer containing 100 mmol/L Tris-HCL (pH 6.8), 20% (*v*/*v*) glycerol, 8% (*w*/*v*) SDS, 5% β-mercaptoethanol, and 0.05% bromophenol blue. Tris-Tricine SDS-PAGE, using 4% stacking gel (29:1 acrylamide-bisacrylamide, pH 8.45 1 mol/L Tris-HCl, and 0.1% SDS), and 16% separating gel (29:1 acrylamide-bisacrylamide, pH 8.45 1 mol/L Tris-HCl, 0.1% SDS, and 10% *v*/*v* glycerol), was used to separate the reaction products (Fig. 9A). Phosphorimager (Molecular Dynamics, Storm 860) was used to capture the images. The image file was applied in IMAGEQUANT TL (GE Healthcare) software to detect the strand product and the relative inhibitory activity (Fig. 9B).

### Leading compound validation and further pharmacological research

The process of finding a new drug against a chosen target for a particular disease usually involves high-throughput screening (HTS), wherein large libraries of chemicals are tested for their ability to modify the target. Another important function of HTS is to show how selective the compounds are for the chosen target. The ideal is to find a molecule which will interfere with only the chosen target, but not other, related targets. To this end, other screening runs will be made to see whether the “hits” against the chosen target will interfere with other related targets—this is the process of cross-screening. All these processes will require several iterative screening runs. Once a lead compound series has been established with sufficient target potency and selectivity and favorable drug-like properties, one or two compounds will then be proposed for drug development. The best of these is generally called the lead compound.

After the leading compound with reasonable inhibition to virus proliferation with clear mechanism could be identified from the comprehensive antiviral discovery system described above, we pass through our leading compound to the chemists to modify the chemical structure and re-cycle these new compounds in our system to further validate the improvement to the inhibition effect and lower the cytotoxicity. Once a leading compound can be generated with good potency and low cytotoxicity, it will be transferred to pharmacologist to verify the pharmacological features, including the bioavailability, pharmacokinetic property, etc. We have to notify that although the finding of a leading compound with potent inhibition effect to virus, the ratio to produce a final antiviral drug is still very limited due to the poor pharmacological features and we have to keep a long way in the battle with virus.
